# Analysis of the Urine

**Published:** 1886-10

**Authors:** 


**Analysis of the Urine**, with Special Reference to Diseases of the Urinary Apparatus. By M. B. HOFFMANN, Professor in the University of Gratz; and R. ULTZMANN, Tutor in the University of Vienna. Second enlarged and improved edition. 8vo. Cloth, $2.00. New York: Appleton & Co., 1, 3 and 5 Bond street.

This admirable work is translated by Drs. Brune and Curtis. The first edition called forth, the unqualified praise from the medical press, and we are glad to see a new edition, for we believe it to be one of the best guides to the diagnosis of diseases affecting the urinary apparatus. The plates which accompany it are excellent.

